# Enhancing drug checking services for supply monitoring: perspectives on implementation in syringe service programs in the USA

**DOI:** 10.1186/s12954-023-00924-5

**Published:** 2024-01-13

**Authors:** Kyle J. Moon, Heather D. Whitehead, Anne Trinh, Kathryn A. Hasenstab, Kathleen L. Hayes, Debra Stanley, Brittany Carter, Rick Barclay, Marya Lieberman, Saira Nawaz

**Affiliations:** 1 Center for Health Outcomes and Policy Evaluation Studies, Ohio State University College of Public Health, 381 Cunz Hall, 1841 Neil Avenue, Columbus, OH 43210 USA; 2 Department of Mental Health, Johns Hopkins Bloomberg School of Public Health, Baltimore, MD USA; 3 Department of Chemistry and Biochemistry, University of Notre Dame, Notre Dame, IN USA; 4 Imani Unidad, Inc., South Bend, IN USA; 5 Equitas Health, Columbus, OH USA; 6 Division of Health Services Management and Policy, Ohio State University College of Public Health, Columbus, OH USA

**Keywords:** Drug checking, Overdose prevention, Fentanyl test strips, Immunoassay strips, LC–MS, Public health, Harm reduction, Drug supply, Implementation research

## Abstract

**Background:**

Shifts in the US drug supply, including the proliferation of synthetic opioids and emergence of xylazine, have contributed to the worsening toll of the overdose epidemic. Drug checking services offer a critical intervention to promote agency among people who use drugs (PWUD) to reduce overdose risk. Current drug checking methods can be enhanced to contribute to supply-level monitoring in the USA, overcoming the selection bias associated with existing supply monitoring efforts and informing public health interventions.

**Methods:**

As a group of analytical chemists, public health researchers, evaluators, and harm reductionists, we used a semi-structured guide to facilitate discussion of four different approaches for syringe service programs (SSPs) to offer drug checking services for supply-level monitoring. Using thematic analysis, we identified four key principles that SSPs should consider when implementing drug checking programs.

**Results:**

A number of analytical methods exist for drug checking to contribute to supply-level monitoring. While there is likely not a one-size-fits-all approach, SSPs should prioritize methods that can (1) provide immediate utility to PWUD, (2) integrate seamlessly into existing workflows, (3) balance individual- and population-level data needs, and (4) attend to legal concerns for implementation and dissemination.

**Conclusions:**

Enhancing drug checking methods for supply-level monitoring has the potential to detect emerging threats in the drug supply and reduce the toll of the worsening overdose epidemic.

## Background

Drug overdose has been the leading cause of injury death in the USA over the past decade [1], inflicting a devastating toll on families and communities across the country. The overdose epidemic, a public health crisis, has claimed the lives of roughly one million Americans since 1999 [2], with sharp, unprecedented increases since 2019 due to the emergence—and proliferation—of synthetic opioids, namely fentanyl and its analogues [1, 3, 4]. The potency and ubiquity of synthetic opioids in the drug supply have shifted the risk environment for people who use drugs (PWUD) [3], as stimulants and opioids adulterated with fentanyl have become increasingly pervasive, heightening concerns and anxieties related to overdose risk among PWUD [5, 6]. Recently, the increasing presence of xylazine, a veterinary anesthetic, in drug overdose deaths presents an emergent threat, leading to severe soft tissue damage and potentially heightened overdose risk [7, 8]. Additionally, novel benzodiazepines have emerged in the unregulated drug supply in North America [9, 10], raising concerns about heightened overdose risk.

Amidst notable supply shifts observed over the course of the COVID-19 pandemic [11], drug checking services have been proposed as a crucial public health response to the overdose epidemic in the USA [12–16]. Drug checking services offer a promising strategy to improve knowledge and agency among PWUD navigating the opaque drug market [17, 18]. Fentanyl test strips (FTS), which are used to detect the presence of fentanyl, are widely used among PWUD to make informed decisions about use and mitigate risks [19]. Existing evidence demonstrates that FTS may modify how individuals intend to use, prompting individuals to discard their sample or practice harm reduction techniques [12], such as using a tester shot, using less, using in the presence of others, using more slowly, or ensuring naloxone is accessible [14, 16, 20–22].

FTS and other rapid immunoassay test strips (e.g., benzodiazepine test strips) are commercially available and distributed by many syringe service programs (SSPs) and other harm reduction organization across the USA [23]. Xylazine test strips are currently sold by BTNX, motivated by the needs expressed by PWUD and clinicians alike [8, 24], while critically important tools, rapid immunoassay test strips have noteworthy limitations, suffering from low limits of detection and interferences from adulterants [23]. In providing a binary result (positive or negative), rapid immunoassay test strips provide no information on concentration, which is important for dosing, especially in a market saturated with fentanyl [18]. PWUD have shared that fentanyl is ubiquitous and difficult to avoid [18], thereby limiting the utility of tests to screen for the presence of fentanyl without knowing the concentration of fentanyl in the sample. Additionally, test strips are specific to one substance, or to several compounds of the same class [19]. In other words, an individual wanting to test their sample for fentanyl and benzodiazepines would have to use two strips: one for fentanyl and one for benzodiazepines.

To address these limitations and to offer more detailed analytical data, various harm reduction organizations have piloted the use of Raman spectroscopy and Fourier-transformed infrared (FTIR) spectrometers for drug checking [17, 25, 26]. These devices can be optimized to provide information on the presence and approximations of the amount of multiple compounds simultaneously but typically require users to employ spectral libraries for accurate, routine analysis and are less sensitive than rapid immunoassay test strips [17, 25, 26]. To offset limitations of each analytical method [27], some harm reduction programs use integrated approaches (e.g., using rapid immunoassay test strips in combination with FTIR) [26, 28].

Advances in drug checking are underway, providing potentially life-saving services for PWUD [25], by enhancing market monitoring capacity. Results from drug checking services are often shared within social networks to share information about drug quality with peers but can also feed into public health data systems [29], aiding in the detection of novel adulterants in the supply [21, 30, 31].

In this manuscript, we cast attention to the requirements and considerations of drug checking services for supply-level monitoring. This work was informed by the ongoing collaborations between academic institutions, SSPs, and community partners, and we begin with an overview of the various methodologies proposed, followed by a set of guiding principles that emerged from our discussions of implementation. While drug checking services are implemented across Europe, Australia, and Canada [21, 32, 33], the considerations presented herein were focused on implementation in the US context, particularly within SSPs. The overarching aim is to describe how drug checking services at harm reduction organizations can be used for supply-level monitoring amidst rapid shifts in the drug landscape without compromising individual-level information for PWUD, and in this way, inform public health interventions for the worsening overdose crisis in the USA

## Methods

As a group of public health researchers, analytical chemists, evaluators, and harm reductionists, we used a semi-structured guide to facilitate discussion on key priorities for drug checking services, considering implementation, data, and public health significance. Four possible methodologies were discussed, each of which would be integrated into a SSP. Following the discussion, we conducted a thematic analysis to identify salient themes. These findings were contextualized with extant literature and were further validated by all members of this collaborative and other harm reductionists and public health professionals in Ohio.

## Overview of low-barrier methodologies

**Fig. 1:**
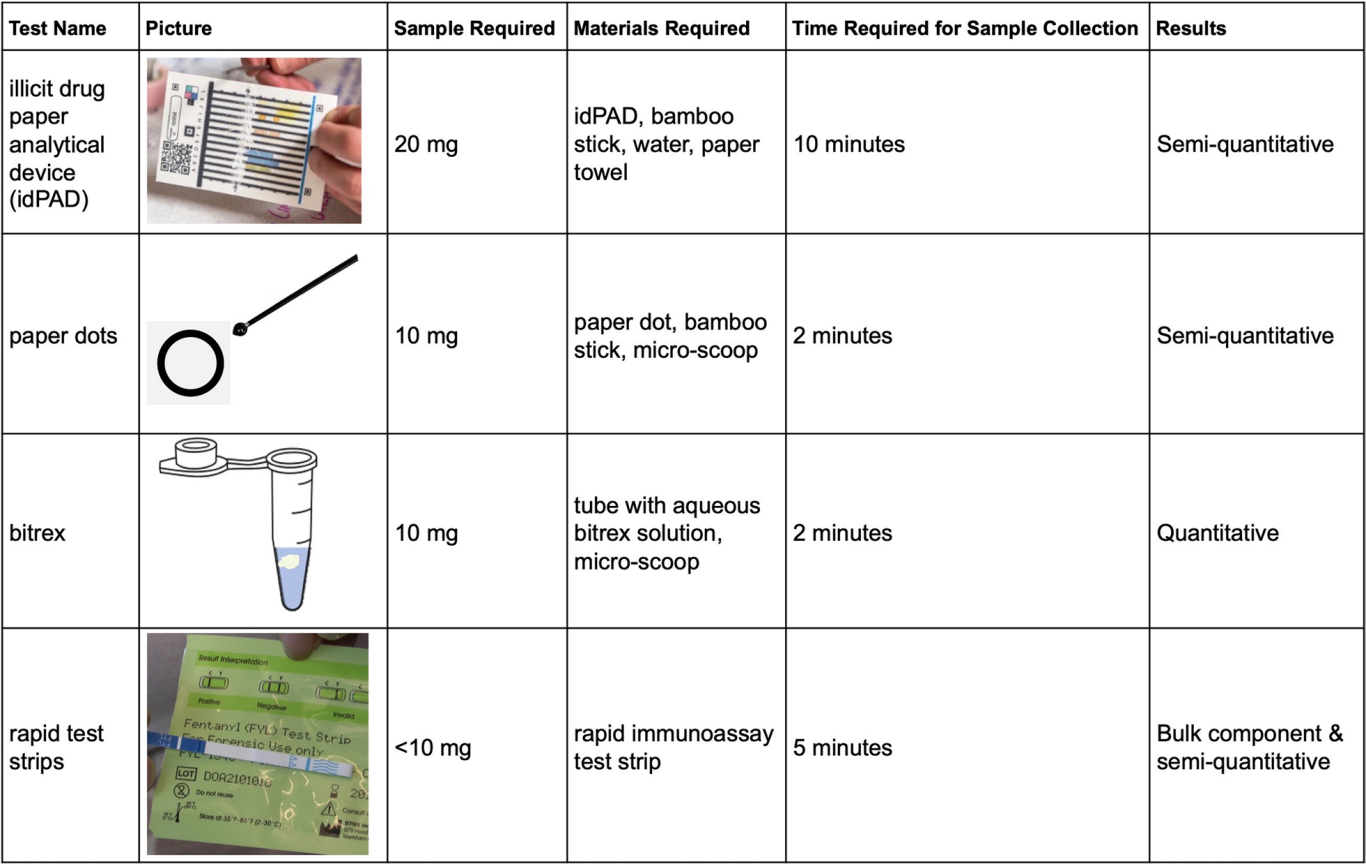
Summary of four low-barrier methods for drug checking services discussed for implementation in SSPs

Drug checking devices, such as the TruNarc Raman spectrometer and Bruker Alpha FTIR [26], provide detailed information for PWUD, but widespread implementation is constrained by legal complexities as well as additional cost and labor requirements for already-stretched harm reduction organizations [19, 25]. All methodologies discussed (Fig. 1) were low-barrier methods, in the sense that minimal materials, costs, and labor would be required for implementation. In this community-academic collaborative, drug checking services would be implemented at the SSP, and with prepaid shipping materials, SSP staff would send completed test materials to the research partner, who would perform all analyses using liquid chromatography with tandem mass spectrometry (LC–MS/MS), a highly selective and sensitive analytical tool for pharmaceutical and illicit drug analysis [23]. Evaluation partners in this collaborative would be responsible for dissemination, feeding results into data streams used by PWUD and public health agencies alike; this is discussed in greater detail in the subsequent section.

The first test makes use of the illicit drug paper analytical device (idPAD) [34], a paper test card developed for the analysis of solid illicit drug samples. To use the cards, solid sample is applied to the card, and the card placed in water to run twelve colorimetric tests, each designed for detecting different functional groups of compounds present in illicit drugs [34]. At present, the idPAD is a useful tool for the analysis of bulk (percent-level) composition of illicit drugs, though it is unable to offer immediate information on drug content to non-trained users. Refinements of the idPAD are ongoing, and a mobile app is now available. The ultimate goal of this app is to capture idPAD images and use a trained neural network to detect the presence of various compounds, adulterants, and cutting agents to provide immediate information on drug content without the need for a trained user [35]. In addition to these developments in progress, the idPAD has been shown to be a useful tool for the collection and analysis of small quantities of illicit drugs for downstream (LC–MS/MS) analyses [23].

The second test takes the same approach as the idPAD but requires minimal time and sample. Individuals press a small mass of sample (10 mg) on an absorbent paper dot with a wax-printed boundary that helps localize and keep the sample in place during transit. Upon receipt of the paper dot, the testing laboratory can extract the solid drug from the paper dot for downstream analysis methods. In the third approach, the same sample mass (10 mg) is placed into a liquid-filled tube containing an aqueous solution of Bitrex, a non-toxic, bittering agent commonly used to prevent ingestion of cleaning products by children. The sample can be directly analyzed with LC–MS/MS. Each of these approaches yields quantitative information (i.e., concentration) after analysis but provides no information for PWUD at the point-of-use.

The final proposed testing method allows for both the generation of rapid data at the point-of-use and for downstream analysis by making use of the commonly employed rapid immunoassay strips (e.g., FTS). With this approach, individuals use fentanyl or benzodiazepine test strips as normal, receiving a rapid dichotomous result (positive or negative). Rather than discarding the used strip, however, it would be sent for downstream analysis, by extraction of illicit drugs from the paper test card [36].

## Key principles

In weighing the strengths and limitations of each testing method, our interdisciplinary team reached a consensus on four guiding principles, or considerations, for selecting a method and implementing drug checking services for supply-level monitoring: (1) immediate utility to PWUD, (2) integration into SSP workflow, (3) balancing individual- and population-level data needs, and (4) attention to the legal context, each of which is described in further detail. Overall, the selected approach should align with the needs and concerns expressed by PWUD.

### Immediate utility to PWUD

Of the four tests discussed, only one method, the rapid immunoassay test strips, provides immediate results to the participant. This was deemed to be of utmost importance because supply-level data cannot come at the expense of individual-level information, especially when such information can be used to inform decision-making related to use and, ultimately, reduce overdose risk [12, 14, 16, 20, 21]. In the final three tests, small amounts (10 mg) of sample are required. The idPAD, in contrast, requires much larger amounts (20 mg), presenting a significant barrier to implementation. Demonstration of immediate benefit to PWUD will be key in building trust among prospective participants.

### Integration into SSP workflow

Considerations of the operational context were critical in thinking about the feasibility of implementation at the SSP. The time required for the idPAD would interfere with the existing SSP workflow, as there are often space constraints and lines of people waiting to enter during operating hours, although resources and structures vary widely between SSPs [37, 38]. The processes for the second test using paper dots were cumbersome, often requiring assistance and a flat surface. The ease of the third test, in which individuals simply placed a scoop of sample into a liquid vial or tube, made it a feasible option. Similarly, FTS are portable, meaning they are already distributed by SSPs for use off-site, causing no changes to existing processes.

Since FTS are already distributed by most SSPs, no disruptions would be made to SSP operations. Additionally, advancements in harm reduction are underway in Ohio with the installation of public health vending machines (PHVMs) [39]. PHVMs are stocked with a range of essential supplies for PWUD to mitigate drug-related harms, including but not limited to sterile injection equipment, HIV test kits, condoms, sharps containers, naloxone, and FTS [39]. FTS included in PHVMs could include prepaid mailing materials and information about the testing service, where rather than discarding the used strip, individuals submit the strip for analysis to contribute to supply-level monitoring [23].

**Fig. 2:**
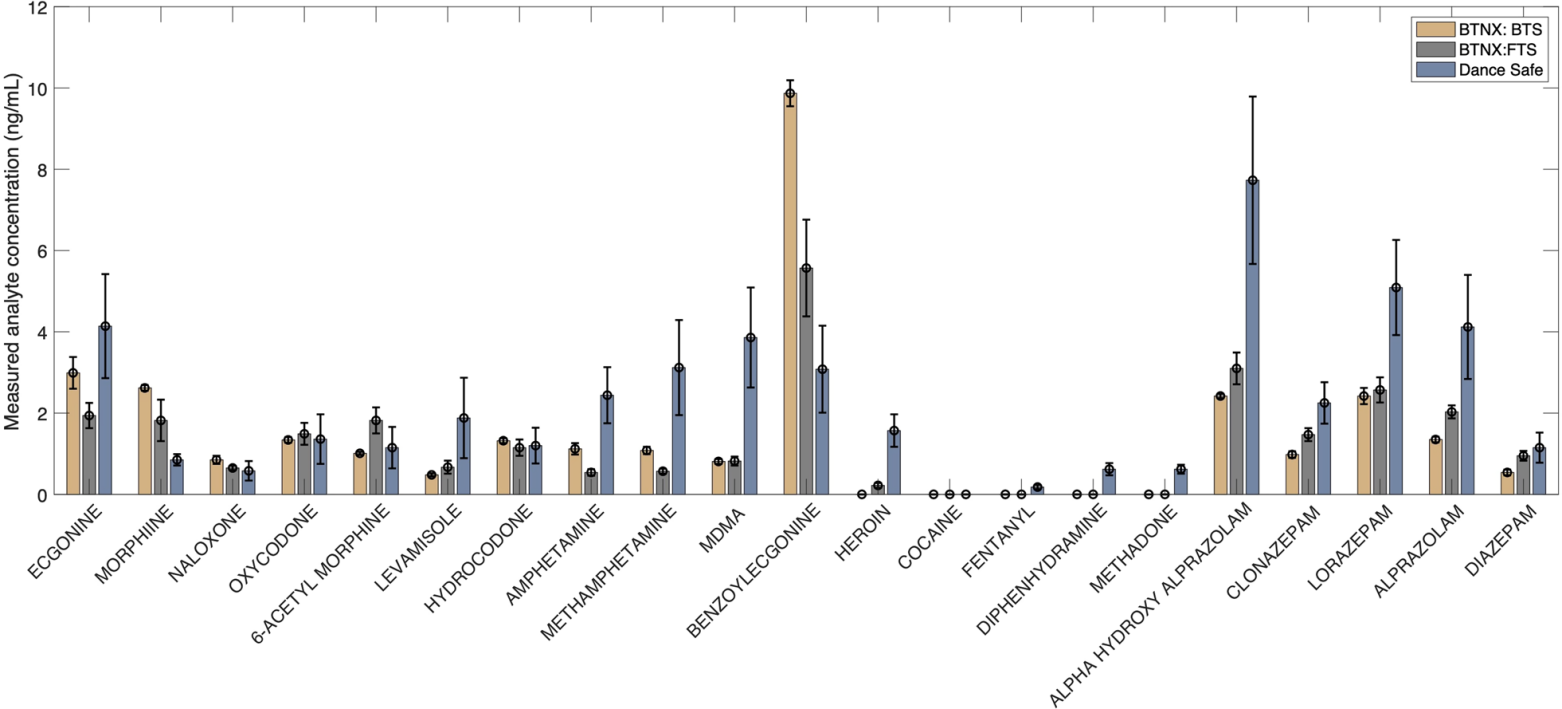
Three FTS (two lots manufactured by BTNX, one lot manufactured by DanceSafe) were run following manufacturer guidelines with aqueous solutions of 5000 ng/mL of each of the drugs or drug metabolites. Each strip was dried, stored for a week, then extracted with 5 mL of water/methanol 9:1 with sonication. Analysis of the amount of drug or drug metabolite that was extracted from the strip into the water/methanol solution was performed as previously described [23]

As an example of a potential downstream analytical method, the Lieberman group has developed sensitive tandem LC–MS/MS analysis for 22 common drugs and drug metabolites [23]. The limit of detection for all analytes is below 0.07 ng/mL, and preliminary results show that a wide range of illicit compounds can be recovered from used FTS using this method (Fig. 2). All 21 drugs were recovered above the limit of detection, demonstrating the potential to obtain much more detailed information about the community drug supply than the result that FTS provide at the point-of-use. The current drug market has been characterized by fentanyl ubiquity [18], and thus, there will likely be shifts in demand for alternative test strips (e.g., xylazine test strips), as opposed to FTS. The method described herein is not limited to FTS, meaning used xylazine test strips could also be used for downstream analysis, but further work is needed to assess how drug-specific antibodies (e.g., fentanyl-specific antibody on FTS) affect the recovery of different drugs. Additionally, future studies should assess how long different drugs can be stored on used immunoassay test strips, how effectively and consistently they can be removed for analysis, and whether other drugs or cutting agents interfere with recovery or downstream analysis.

Besides used test strips, other drug paraphernalia (e.g., cookers, cottons, bags) could be analyzed by extracting residue, but PWUD would receive no information at the point-of-use. This may be a beneficial approach for SSPs and harm reduction organizations that have working relationships with local law enforcement and prosecutors for safe disposal of syringes. For example, when law enforcement officials in St. Joseph County, Indiana, find used drug paraphernalia (e.g., syringes, cookers) in the community, they contact employees from the local harm reduction organization to safely collect and dispose of such materials. Paraphernalia collected for disposal, with the exception of syringes, could be submitted for analysis to contribute to supply-level monitoring. While there are previous studies where syringes were used for analysis [40], this approach requires safeguards for safe transport and handling of biohazardous materials. Additionally, submitting used syringes would limit analyses to substances consumed by injection, whereas collecting test strips or paraphernalia other than syringes accommodates testing of substances that were consumed through various routes of administration. This is an important consideration, as snorting has become increasingly common in the synthetic opioid era [41–43].

### Balancing individual- and population-level data needs

**Fig. 3:**
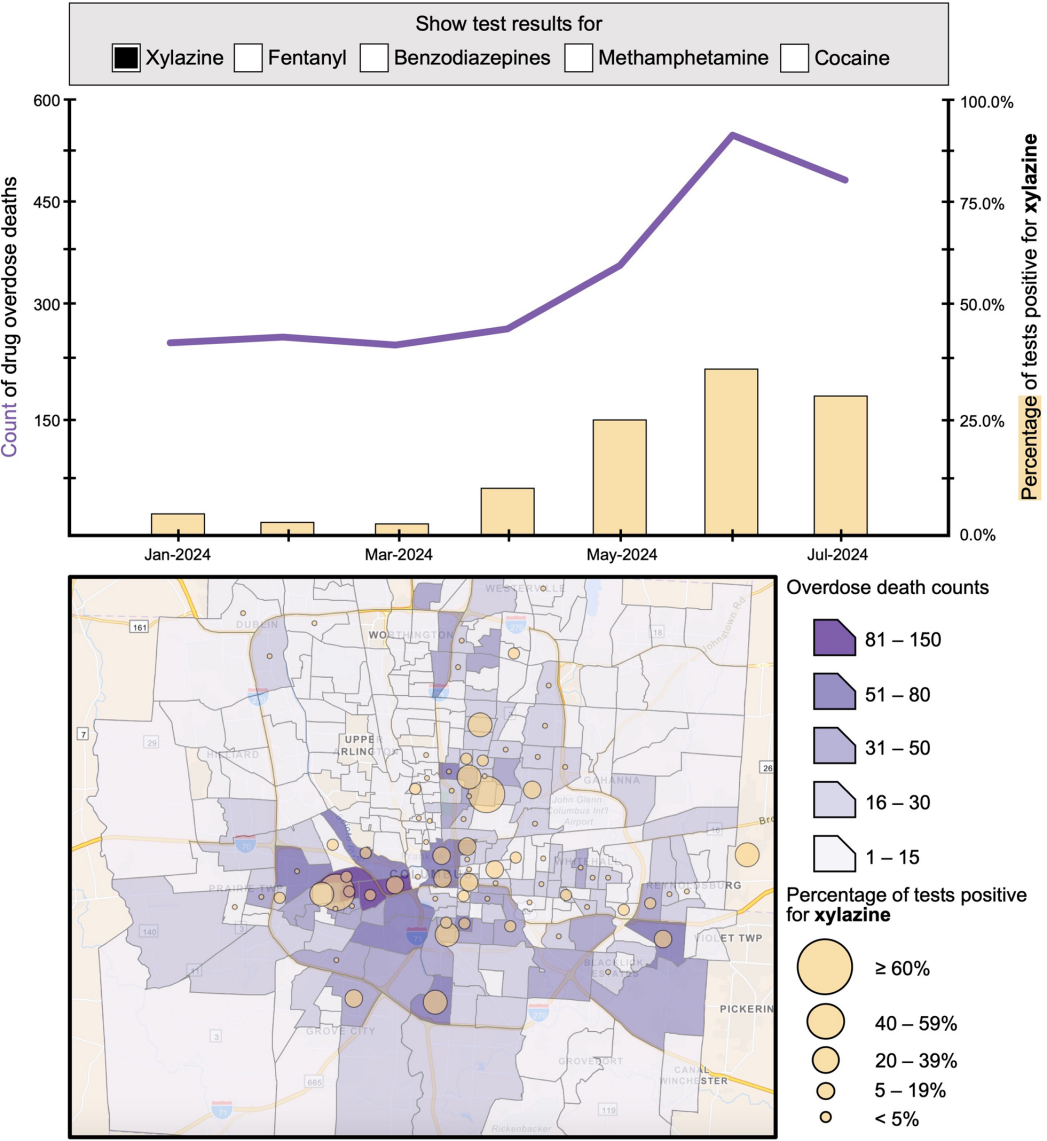
Mock dashboard of overdose trends overlaid with supply-level monitoring. Data were constructed to provide an example of how drug checking data can be superimposed on overdose dashboards to assess geospatial and temporal trends to better understand associations between supply shifts and overdose risk [46, 47]

Members of this collaborative discussed the importance of utilizing existing infrastructure for dissemination of results to ensure that, even if there is a data lag, the results are useful and relevant to PWUD in the community. For example, results can feed into “bad batch alerts” systems. The SOAR (Safety, Outreach, Autonomy, Respect) Initiative in Ohio has developed a mobile application, modeled after a text messaging service in Baltimore [44, 45], that alerts PWUD when overdoses have surged and when fentanyl has been detected and reported in multiple batches in a particular geographic area. Feeding results into a data stream that is trusted and used by PWUD maximizes the utility of data. Beyond bad batch alerts, this information can be used by SSP staff to share information with participants, effectively tailoring information to current supply trends. Similarly, public health departments often manage dashboards to monitor and evaluate overdose data; such dashboards can be complemented by overlaying overdose trends with supply-level trends (Fig. 3), facilitating the detection of emergent shifts and threats.

While aggregate data can provide important information for supply-level monitoring, providing anonymous individual-level data can maximize benefits to individuals participating in drug checking programs. The dashboard (streetsafe.supply) developed and maintained by the Injury Prevention Research Center at the University of North Carolina–Chapel Hill, which offers mail-based drug checking services, is one exemplar [48]. Each sample is assigned an anonymous ID, which individuals make note of prior to submission. Individual results are posted to the dashboard with the associated sample ID, allowing individuals to access the results from their sample. Publishing individual-level results on a dashboard underscores the need to protect participants’ anonymity to avoid both (a) criminalization [49] and (b) retaliation from those who sell drugs for perceived “snitching”, [50, 51] potentially disrupting supply chains or social networks [52].

### Attention to the legal context for implementation and dissemination

Recognition of the legal complexities associated with each approach was also central to the discussion. Asking individuals to provide a sample on-site requires significant trust [25], and in most states, drug possession on SSP premises is prohibited [19], meaning individuals would have to complete the test off-site and bring completed materials at their next visit. Alternatively, the SSP could provide individuals with prepaid mailing supplies, allowing individuals to complete and submit the test off-site simultaneously. Whether SSP participants or staff are responsible for mailing completed testing materials is of consequence to the research partner because staff can ship materials according to a planned schedule, whereas samples ready for analysis will be received sporadically when submitted by individual participants.

The level of data collected and reported should be scrutinized, carefully considering the utility of such information to PWUD as well as how such information could be used by police. At minimum, prospective participants should be fully informed on how data will be used for supply-level monitoring. Scholars have raised concerns about police using supply-level monitoring—and geospatial data, in particular—to target enforcement resources [49]. Protecting participants’ anonymity is paramount to ensure public health monitoring does not facilitate increased—and counterproductive—criminalization among individuals participating in harm reduction programming [49].

Drug paraphernalia laws can prevent PWUD from participating in harm reduction programming [53], and thus, may present a barrier to participation in drug checking services. Paraphernalia laws broadly prohibit the possession of equipment that is associated with illicit drugs, even equipment used for testing, although considerable heterogeneity exists across states [19, 53], and the legal status of FTS has often been ambiguous [53]. In 2021, the Centers for Disease Control and Prevention (CDC) and the Substance Abuse and Mental Health Services Administration (SAMHSA) announced new regulations that now allows federal funding to be used to purchase FTS. Historically, in as many as 30 states, it was illegal to *possess* drug checking equipment, which included FTS, and 33 states prohibited the *distribution* of drug checking equipment [19]. Penalties for violation of drug paraphernalia laws varied widely, ranging from civil fines to multi-year sentences [19]. Even though regulations have changed, and loopholes exist [54], limited awareness may discourage participation and implementation of drug checking programming due to concerns about potential criminalization [53], underscoring the need to promote awareness among PWUD. Furthermore, there are complexities associated with new regulations that still limit participation in the full range of harm reduction services. For example, in Ohio, the recent passage of SB 288 excludes *only* FTS from drug paraphernalia laws [55]; rapid immunoassay test strips for other scheduled substances would still be subject to drug paraphernalia laws.

Drug paraphernalia laws are particularly relevant for partners collecting and submitting used paraphernalia for analysis. This approach requires strong working relationships between harm reduction organizations and local law enforcement, which can be facilitated by providing officers with training and resources that detail the well-established benefits of harm reduction services to PWUD—and the community at-large [56]. These relationships, or even partnerships, between harm reduction organizations and law enforcement are critical because officers have discretion in how they respond to, and enforce, substance use-related incidents [57–60].

## Processes to accelerate implementation

In addition to considerations for implementation at SSPs and with PWUD, special considerations exist for the implementation of these protocols at academic research institutions conducting downstream analyses of illicit compounds. While analytical reference solutions of controlled substances can be purchased and handled by academic researchers without additional approvals, the purchasing, handling, and disposals of solid illicit drug standards and samples are regulated by government entities at the federal (Drug Enforcement Administration [DEA]), state (State Pharmacy Boards), and local levels. Specifically, academic laboratories wishing to work with solid illicit drugs are required to acquire the license(s) for the schedules of drugs of interest. It is unclear, however, that these regulations apply to *used* FTS, as they are garbage and do not require special protocols for waste disposal. In any case, approvals and documents of support or acknowledgment from government organizations, especially the DEA, may facilitate increased stakeholder support, alleviating concerns about legality and enforcement. Additionally, forming working relationships between harm reduction organizations and local law enforcement can help safeguard PWUD, mitigating concerns about policing and criminalization of those participating in drug checking and other harm reduction services [56, 57, 59, 60]. If applications or standard operating procedures are required, these should be initiated as early as possible to enable timely incorporation of samples collected through SSP collaborations.

Collaborations with academic laboratories and SSPs provide an opportunity to develop and validate methods for targeted and non-targeted analysis, which depend on real-world samples because adulterants in the supply can cause chemical interference that would not be observed when tested with pure, analytical-grade compounds. SSPs can provide academic institutions with diverse, real-world samples that enhance the utility of novel tests and technologies, while academic institutions provide access to analytical instrumentation (e.g., LC–MS/MS) that facilitate robust, detailed analyses for drug checking, overcoming the limitations of existing rapid tests and advancing supply-level monitoring efforts [25].

## Implications for public health policy and practice

The USA faces a worsening overdose crisis, exacerbated by supply shifts and the emergence of xylazine, altering the risk environment for PWUD [3, 7, 8]. In the absence of safe supply, drug checking services are an urgent need [12, 13], as these services provide PWUD with agency to navigate an unpredictable drug market [18]. Many SSPs and harm reduction programs distribute rapid immunoassay test strips, and community-academic partnerships provide a promising avenue to enhance existing drug checking services for supply-level monitoring, by developing and validating methods for analysis (e.g., xylazine test strips).

A wide variety of technologies exist that can be applied for drug checking services [17, 26, 27], each of which has its own strengths and limitations. Faced with budgetary constraints, harm reduction organizations will have to balance tradeoffs, and although there is likely not a one-size-fits-all approach, the implementation of drug checking services should be guided and informed by key principles. For one, tests should prioritize immediate utility to participants. Additionally, the dissemination of results should carefully balance individual- and supply-level information needs, while ensuring anonymity to mitigate the potential for targeted policing and criminalization among participating individuals and communities [49]. The processes for dissemination should also be considered, looking to existing, trusted data infrastructure used by PWUD (e.g., bad batch alert systems) to maximize the utility of data.

Existing supply monitoring efforts are limited and typically stem from law enforcement seizures and postmortem toxicology results, both of which are subject to selection bias [13]. In the collaborative described herein, SSPs will continue to distribute FTS as normal, but participants can submit the used test strip for analysis rather than discarding it. This approach ensures participants receive immediate results that can inform how they use, while also contributing to supply-level data. The costs associated with testing present a barrier to the scale and sustainability of community–academic partnerships—and to drug checking services more broadly. Opioid settlement funds may provide one mechanism to fund drug checking and other essential harm reduction services that have long been the financial responsibility of community-based organizations [61].

## Conclusions

Drug checking services are potentially life-saving interventions, promoting agency among PWUD to mitigate risks in an unpredictable environment. Augmenting existing drug checking programs to facilitate supply-level monitoring has the potential to detect emerging threats in the drug supply, and in this way, public health agencies can proactively respond to supply shifts and tailor interventions to curb the toll of the overdose epidemic.

## Data Availability

Not applicable.

## Abbreviations

FTS: Fentanyl test strips
FTIR: Fourier-transformed infrared spectroscopy
idPAD: Illicit drug paper analytical device
LC–MS/MS: Liquid chromatography with tandem mass spectrometry
PHVM: Public health vending machines
PWUD: People who use drugs
SSP: Syringe service program
